# Peripartum cardiomyopathy: a global effort to find the cause and cure for the rare and little understood disease

**DOI:** 10.1007/s12551-022-00930-0

**Published:** 2022-01-24

**Authors:** Amy Li, K. Campbell, S. Lal, Y. Ge, A. Keogh, P. S. Macdonald, P. Lau, John Lai, W. A. Linke, J. Van der Velden, A. Field, B. Martinac, M. Grosser, Cristobal dos Remedios

**Affiliations:** 1 Department of Pharmacy & Biomedical Sciences, La Trobe University, Bendigo, VIC Australia; 2 Centre for Healthy Futures, Torrens University, Pyrmont, Australia; 3 Sydney Heart Bank, University of Sydney, Sydney, Australia; 4 Department of Physiology, University of Kentucky, Lexington, KY USA; 5 Department of Cell and Regenerative Biology, Department of Chemistry, Human Proteomics Program, University of Wisconsin-Madison, Madison, WI USA; 6 Heart Transplant Unit, St Vincent’s Public Hospital, Victoria St, Darlinghurst 2010, Sydney, Australia; 7 University of New South Wales, Kensington, Australia; 8 St Vincent’s Hospital Cardiology, Darlinghurst, Australia; 9 Victor Chang Cardiac Research Institute, 405 Liverpool St, Darlinghurst, Australia; 10 Australian Genome Research Facility, Melbourne, Australia; 11 Australian Genome Research Facility, Brisbane, Australia; 12 Clinic for Cardiology and Pneumology, University Medical Center, Göttingen, Germany; 13 VU University Medical Center, Amsterdam, The Netherlands; 14 St Vincent’s Hospital Pathology, Darlinghurst, Australia; 15 23Strands, 107 Pirramina Rd, Pyrmont, Australia

**Keywords:** Peripartum cardiomyopathy, Gene mutation in cardiomyocytes, Heart failure in pregnancy

## Abstract

In this review, we present our current understanding of peripartum cardiomyopathy (PPCM) based on reports of the incidence, diagnosis and current treatment options. We summarise opinions on whether PPCM is triggered by vascular and/or hormonal causes and examine the influence of comorbidities such as preeclampsia. Two articles published in 2021 strongly support the hypothesis that PPCM may be a familial disease. Using large cohorts of PPCM patients, they summarised the available genomic DNA sequence data that are expressed in human cardiomyocytes. While PPCM is considered a disease predominately affecting the left ventricle, there are data to suggest that some cases also involve right ventricular failure. Finally, we conclude that there is sufficient evidence to warrant an RNAseq investigation and that this would be most informative if performed at the cardiomyocytes level rather than analysing genomic DNA from the peripheral circulation. Given the rarity of PPCM, the combined resources of international human heart tissue biobanks have assembled 30 ventricular tissue samples from PPCM patients, and we are actively seeking to enlarge this patient base by collaborating with human heart tissue banks and research laboratories who would like to join this endeavour.

## What is peripartum cardiomyopathy (PPCM)?

Literally translated, PPCM means: *peri*- (around the time of), *partum* means birth or delivery, *cardio* refers to the heart, *myo*- means muscle and *pathy* meaning disease. Therefore, it is a disease that affects the heart muscle that occurs either late in pregnancy, or within 6 months following delivery. However, it is not uncommon for the onset of heart failure to present up to 6 months post-partum. PPCM is life-threatening and despite being the largest contributor to pregnancy-related heart failure, the cause remains unknown, i.e. idiopathic (Arany and Elkayam 2016).

## Incidence and risk factors

In a recent review, Honigberg and Givertz (2019) reported that while the incidence is low, it differs markedly worldwide. In China it is about 1:350 live births (Fett et al. 2005), in the USA (Kolte et al. 2014) and in South Africa (Desai et al. 1995) it is about 1–2:1,000 live births and in Japan it is about 1:20,000 live births (Kamiya et al. 2011). In Australia, the incidence is unknown; however, we estimate it to be similar to the USA based on clinical data available at St. Vincent’s Hospital, Sydney.

In the USA, there are marked differences in its incidence, depending on the age of the mother. In 2004, mothers with PPCM aged between 15 and 19, 20 and 29 and 30 and 39 have incidences of 0.5–1:1000 births, compared to 40–54-year-old mothers where the incidence is about 3:1000 live births. A follow-up study 7 years later found the incidence in those older than 40 years old increased to about 4.5:1000 live births (Kolte et al. 2014; Sliwa et al. 2017). The increase may be due in part to improved diagnosis, but also because older maternal age is a risk factor. The major risk factors for the increased incidence of PPCM include hypertensive disorders such as pre-eclampsia, diabetes, obesity and multiple gestations which are associated with in a 9–22% increased prevalence compared to the general population worldwide (Sliwa et al. 2017).

## Clinical presentation and diagnosis

PPCM is potentially a fatal disease with patients rapidly developing an enlarged and weakened contractile heart with a reduced left ventricular ejection fraction (LVEF) of < 45% in the absence of other identifiable causes. It is diagnosed in the third trimester or most commonly within the first 6 months post-partum.

PPCM is not a precisely defined entity (Honigberg and Givertz 2019). The disease not only affects the mother, but it also affects her baby, as well as her immediate and/or extended family who will be needed in the months to care for the baby while the mother is extremely unwell. While most patients respond to conventional medication (see below), some may require mechanical circulatory assistance such as a left ventricular assist device (LVAD) while others may proceed to orthotopic heart transplantation (Rasmusson et al. 2012). The NIH website lists the following symptoms: tachycardia, chest pain, excessive fatigue, tiredness low cardiac output, renal and hepatic dysfunction increased urination (Genetic and Rare Diseases Information Centre 2021).

## Treatments and medications

Amongst women diagnosed with PPCM, 30–50% fully recover on medication but ~ 4% require implantation of a left and/or right ventricular assist device (LVAD) as a “bridge” to heart transplantation (Hu et al. 2013), and 9% will die following transplantation (Keogh et al. 1994). Individual prognosis is worst for patients with the lowest LVEF or severe diastolic dysfunction. Up to 25% of patients with PPCM rapidly develop heart failure and will require orthotopic heart transplantation, which can be highly successful. Interestingly, PPCM patients at St Vincent’s Hospital Heart & Lung Transplant Unit in Sydney who received donor hearts following circulatory death (DCD) remain at the New York Heart Association Classification of Heart Failure (NYHA) class I but with essentially normal biventricular function (Chew et al. 2019).

Medications used to treat this condition include loop diuretics, beta blockers, nitrates, digoxin and others (angiotensin converting enzyme inhibitors, angiotensin blockers such as sacubitril) (Kim and Shin 2017). These drugs broadly reduce fluid accumulation and block the harmful neurohumoral cascade in heart failure. However, some of these medications are incompatible with pregnancy and lactation (Davis et al. 2020).

For non-medically trained readers, Hassanabad et al. (2020) provide a lucid account of the dramatic case of a 35-year-old PPCM patient from the time she arrived at a hospital with severe bi-ventricular heart failure (LVEF < 10%), how her medical history was assessed, how she was differentially diagnosed, investigated and managed as she went through multiple interventions including temporary left ventricular assist support.

## American women of African descent

A recent retrospective review of 220 PPCM patients clearly demonstrated that American women of African descent are significantly worse off than non-African American PPCM patients (Irizarry et al. 2017). In this study, African American women typically presented at a younger age with less than 40% of the population over the age of 30 compared to the 70% of non-African patients in the age bracket above 30 years old. While the diagnosis was made in 90% of both cohorts during the post-partum period, ethnicity resulted in a surprisingly divergent trend of when the initial diagnosis was confirmed. In the non-African cohort, close to half of the diagnoses were made within the first week post-partum, with the numbers dropping off exponentially thereafter. However, in the African American cohort, only about 20% of the diagnoses were made in the first week, and the number of initial diagnoses steadily increases towards the 5-month mark. In the latter group, almost half of those that initially presented with an LVEF < 30% were twice as likely to worsen compared to their non-African American counterparts. Furthermore, those who eventually recovered took nearly twice as long despite comparable treatment regimens (Irizarry et al. 2017). Similarly, Nabbaale et al. (2020) studied 236 PPCM cases in black Ugandan women and reported clinical data (echocardiology, NYHA class III/IV, LVEF ≤ 55%) that were comparable to Irizarry et al. (2017) except the Ugandan cohort reported no maternal or foetal mortality. The underlying cause of the disparity in disease onset and outcomes between ethnicities remains unknown. 

A more recent report by Getz et al. (2021) showed that socioeconomic status is a factor which contribute to the outcomes of African American PPCM patients.

 Additionally, genetic backgrounds and predisposition to specific polymorphisms in women of African descent was also thought to contribute to the incidence of PPCM and subsequent risk factors. In a mix-race cohort of 97 women with PPCM, patients carrying mutations for the TT polymorphism of the GNB3 gene had a lower LVEF, and this functional decline remained at 6 and 12 month postpartum (Sheppard et al. 2016). Surprisingly, the TT polymorphism was found at twice the prevalence in PPCM women of African ancestry compared to women of caucasian descent, and was associated with poorer outcomes at 12 months postpartum. 

## Plasma markers PPCM suggest vascular and/or hormonal cause

An early report based on a mouse model suggested that PPCM may be a disease triggered by placental and pituitary hormones (Hilfiker-Kleiner et al. 2008). They showed that although signal transducer and activator of transcription 3 (STAT3) is not expressed in cardiomyocytes, it is nevertheless involved in a complex set of molecular interactions that involve increased production of cathepsin D, an enzyme secreted by cardiomyocytes. This enzyme cleaves the maternal pituitary-derived nursing hormone prolactin (PRL) producing a 16-kD fragment that induces apoptosis in cardiomyocytes (Hilfiker-Kleiner et al. 2007). In this study, a mouse knock-out of the *STAT3* gene developed a phenotype that included vascular “drop out” in late pregnancy. Importantly, administration of the drug bromocriptine inhibited the secretion of prolactin, which in turn reversed peripartum cardiomyopathy in the mouse. The 16-kD prolactin peptide also triggers endothelial cell apoptosis and secretion of miRNA146a into the circulation, producing dysfunction and apoptosis. Thus, miRNA146a is considered to be a circulating biomarker for PPCM (Halkein et al. 2013).

Circulating Fms-like tyrosine kinase (sFlt) is derived from the placenta and is elevated in preeclampsia, a pregnancy associated complication characterised by hypertension and proteinuria occurring after 20 weeks of gestation in previously normotensive women. sFlt is toxic to the heart and is therefore a potential cause of PPCM (Bello and Arany 2015). It inhibits vascular endothelial growth factor (VEGF), and so leads to the release of nitric oxide, dysfunction and endothelial cell apoptosis. Table 1 summaries the levels of these circulating plasma biomarkers in women with uncomplicated and complicated pregnancies including PPCM.

**Table 1:** Plasma biomarkers that distinguish between peripartum cardiomyopathy, preeclampsia and normal uncomplicated pregnancy (Ersbøll et al 2021). Sample cohort consisted of 28 women per group

Gene	Name	Normal	Pre-eclampsia	PPCM
sFlt-1	Soluble Fms-like tyrosine kinase	63.4	67.6	74.9
PlGF	Placental growth factor	5.8	6	7
CTSD	Cathepsin D	10	8	19
NT-proBNP	N-terminal pro B-type natriuretic peptide	6.9	6.8	13.8

## Cardiomyocyte gene mutations that contribute to PPCM

Goli et al. (2021) performed next generation sequencing (NGS) using 67 genes on a cohort of 469 women (41% of African descent) from multiple centres. These women were retrospectively identified with PPCM characterised by reduced LVEF towards the end of pregnancy or in the months following delivery. They report that 10.4% of these women carried truncating mutations in the giant *TTN* gene, but they also identified over-representation of truncated variants in filamin C (*FLNC*), desmoplakin (DSP) and BAG cochaperone 3 (BAG3) which had not previously been associated with PPCM.

The titin gene (TTN) encodes a sarcomeric protein, the largest known protein to man (dos Remedios and Gilmour 2017). Its N terminus is located in the Z disc of the cardiomyocyte sarcomere and the sequence runs uninterrupted through the I band and half the A band where its C-terminus binds to the C-terminus of another titin molecule from the other half of the sarcomere, a distance of over 1 µm (dos Remedios and Gilmour 2017). Of all the known *TTN* mutations, truncations that occur in titin where it binds to the myosin thick filaments in the A band of the sarcomere are most common (Roberts et al. 2015).

Our understanding and ability to pinpoint causative genetic mutations are still evolving. Goli et al. (2021) identified 70 mutations in 12 genes from PPCM sampling. Another recent study found 21 genes were associated with cardiomyocytes that contribute to the genetic predisposition of PPCM patients (Spracklen et al. 2021). Some of these gene mutations were also reported by Ware et al. (2016) who constructed sequencing libraries of 43 genes and identified 26 truncating variants in 8 genes that were associated with dilated cardiomyopathy. The available published data are summarised in Table 2. However, Ware et al. cautioned that although the data are suggestive, the value of genomic data in determining the prognosis for PPCM patients requires further studies.

**Table 2:** Gene mutations reported in PPCM patients. This list the genes and their corresponding proteins found in cardiomyocytes of peripartum cardiomyopathy. These genes are categorised according to their locations relative to the cardiomyocyte, between the sarcomeres in the sarcoplasm, bound to the cell membranes including the intercalated discs (ICDs), and in or associated with the nuclei. Seventy-seven percent of PPCM patients tested negative for pathogenic mutations. The remaining 23% tested positive for *TTN* (~ 10%), sarcomeric genes (~ 5%), *SCN5A* (~ 4%), *BAG3* (1%), *SYNM* (1%), *DMD* + *LAMP2* (1%) and other genes (1%)

Protein name	Expression in muscle	Cellular location	Gene	PPCM mutation*	Heart disease^	Gene mutation prevalence#	Binding partners	Cellular function	Reference
Sarcomeric									
BCL2 assoc athogene 3	Cardiac	Z disc	BAG3	T	FDCM	1	Hsp70, small Hsps	Co-chaperone, autophagy	Goli et al. 2021
Myomesin 1	Cardiac, skeletal	M line	MYOM1	pE247K	FDCM		Obscurin, titin	Stretch sensor	Marston et al. 2015
Myosin binding protein C3	Cardiac, skeletal	C zone within A band	MYBPC3	M	FDCM, HCM		Actin, myosin, titin	Regulates actomyosin force	Heling et al. 2020
Myosin heavy chain 6	Cardiac, skeletal	A band	MYH6	T, M	HCM, AF	2	Actin, titin	Motor protein	Ware et al. 2016
Myosin heavy chain 7 beta	Cardiac, skeletal	A band	MYCH7	nN16499K, M	FDCM, HCM	2	Actin, titin	Motor protein	Ware et al. 2016
Obscurin	Cardiac, skeletal	A band	OBSCN	pV2161D, M			Titin	T hick fil/myofibrillogenesis	Marston et al. 2015
Troponin C1	Cardiac, skeletal	I band	TNNC1	M			TNNI3, actin	Ca regulation	Spracklen et al. 2021
Troponin I3	Cardiac	I band	TNNI3	pK36Q, M	FDCM		TNNC1	Ca regulation	Marston et al. 2015
Troponin T2	Cardiac	I band	TNNT2	M	HOCM		Tropomyosin	Ca regulation	Spracklen et al. 2021
Tropomyosin	Cardiac	I band	TPM	T			Actin,MYBPC3,TNNT	Strong loss-of-function	Spracklen et al. 2021
Titin	Cardiac, skeletal	From Z disc to M line	TTN	P11181fs T, M		49	LMM, myomesin	Assembly of myosin filaments	Goli et al. 2021
Cytoskeletal									
Lysosomal assoc memb protein2	Cardiac	Sarcoplasmic	LAMP2	T, M	FDCM, HCM		X-linked	Autophagy	Spracklen et al. 2021
Integrin linked kinase	Cardiac	Sarcoplasmic	ILK	T, M	IHD, FDCM	1	SERCA2a	Stretch receptor, remodelling	Goli et al. 2021
Presenilin 2	Cardiac	Z disc	PSEN2	M	FDCM		RyR2	Amyloid, protease, E-C coupling	Spracklen et al. 2021
Parathyroid-like hormone	cardiac	-	PTHL1R	T				Up by low glucose, hypoxia	Horne et al. 2011
Synemin	Cardiac, skeletal	Intermediate filament	SYNM	T	DCM			Regulates PKA and PI3K signalling	Spracklen et al. 2021
Vinculin	Cardiac	Cytoskeleton	VCL	pA413T, T	FDCM, HCM	1		Early onset of PPCM/FDCM	Goli et al. 2021
Membranous									
Desmin	Cardiac	Intermediate filament, ICD	DES	R406W, T	HCM	1		Uncouple desmin from the ICD	Goli et al. 2021
Duchenne muscular dystrophy	Cardiac, skeletal	Sarcolemma	DMD	T	FDCM		X-linked	Mosaic expression: DMD, LAMP2	Spracklen et al. 2021
Desmoplakin	Cardiac	Intercalated disc	DSP	pR1537C, M	FDCM	6		Intercalated disc cell adhesion	Goli et al. 2021
Fukutin	Cardiac, skeletal	Golgi body	FKTN	T, M	FDCM			Glycosylation of a-dystroglycan	Spracklen et al. 2021
Filamin C	Cardiac	Intercalated disc	FLNC	T		4	ICD proteins	Proto-oncogene, arrhythmias	Goli et al. 2021
Guanine nucleotide-binding protein B3	Cardiac (weak)	Sarcolemma	GNB3	C825T, M				Risk factor, coron art disease	Sheppard et al. 2016
Voltage gated K-channel	Cardiac	G-family channels	KCNH2	T	Long QT			Cardiac conduction defect	Kepenek et al. 2020
Phospholamban	Cardiac	Ca signalling	PLB	p.R9C, M	DCM, HCM		SERCA2a	Calcium signalling	Fish et al. 2016
Plectin	Cardiac, skeletal	Intercalated disc	PLEC	M	AF	1	Tubulin	Intermed fils, MTs, actin fils	Milan 2017
Na voltage-gated channel a subunit 5	Cardiac	Sarcolemma	SCN5A	pS216L, M				Sodium channel	Spracklen et al. 2021
Nuclear									
Laminin subunit alpha 2	Cardiac, skeletal	Basal lamina	LAMA2	pV18981 M		1		Cardiac hypertrophy	Marston et al. 2015
Lamin A/C	Cardiac	Nuclear lamina	LMNA	T	FDCM			Transcriptn, signal transdn	Glöcklhofer et al. 2018
RET (proto-oncogene)	Cardiac	Golgi	RET	M				Proto-oncogene	Spracklen et al. 2021
Thymopoietin	Cardiac	Nuclear lamina	TMPO	T		1			Goli et al. 2021

^*^T, truncating mutation; *M*, missense mutation

^In addition to PPCM

^#^Prevalence of gene mutations found in the populations referenced in far right column

**Fig. 1:**
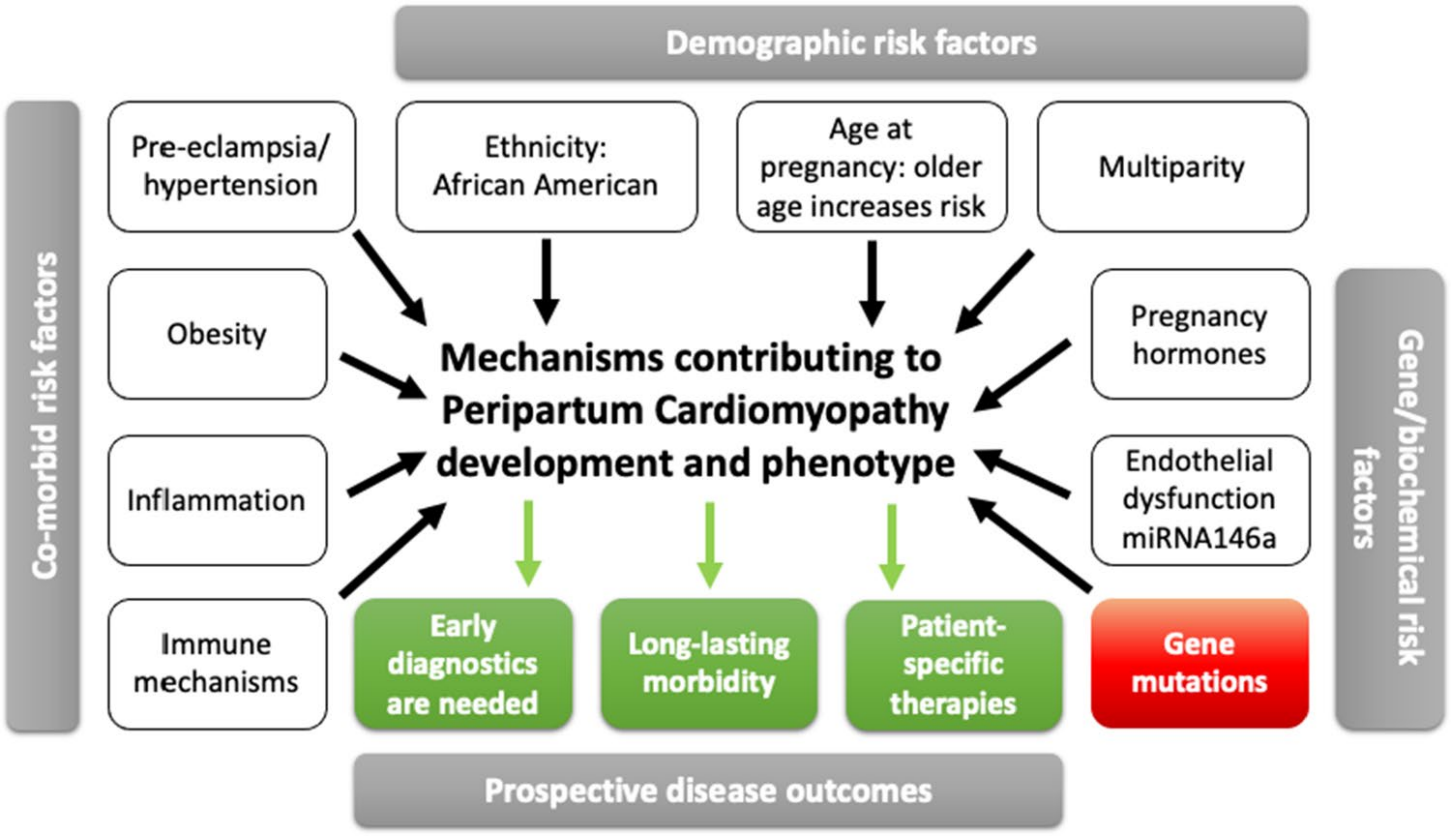
A simplified summary of the risk factors that contribute to peripartum cardiomyopathy. The texts in the white boxes identify risk factors that contribute to the PPCM phenotype and are discussed in detail in the main text. The green boxes represent the outcomes of the PPCM disease. The central theme of this review is a focus on the potential genes that may cause PPCM (red box), discussed in more detail in Table 2

Figure 1 summarises the above discussion of the factors and highlights in red the role played by gene mutations in cardiomyocytes, which is the main thrust of the remainder of this review.

## Is PPCM a variant of dilated cardiomyopathy?

Like idiopathic dilated cardiomyopathy (IDCM), PPCM is a diagnosis of exclusion. Even when the LVEF is < 45%, the left ventricle may not be dilated (van Spaendonck-Zwarts et al. 2014). There is mounting evidence that PPCM is a familial disease that mainly impacts cardiomyocytes and, in several respects, it resembles familial dilated cardiomyopathy (FDCM) (Ware et al. 2016).

## Right ventricular failure in PPCM

Almost without exception, the majority of publications on PPCM refer to left ventricular failure, and although these papers do not specifically exclude right ventricular (RV) failure, the involvement of RV dysfunction has largely been ignored. A search of PubMed revealed at least one report on RV failure by Haghikia et al. (2015) who used cardiac magnetic resonance (CMR) to image the right ventricle in 34 patients with acute PPCM. They were diagnosed using CMR imaging days after delivery, and again within 3 days of developing acute heart failure. Mean LVEF was < 35% in two thirds of the patients. Thirty-five percent of the patients also had a reduced RV ejection fraction function below 40% capacity. Moreover, patients with reduced RV function had more marked LV dilatation. LV was dilated in 91% and RV was dilated in 24% of the patients. Fifty-nine percent of all patients recovered completely. Haghikia et al. (2015) pointed out that although echocardiography provides a very good estimate of LV size and function, CMR can more accurately quantify both LV and RV structure and function. They also used late gadolinium enhanced MR to assess myocardial oedema and scar tissue. All the patients in this cohort were of German or European extraction, but similar RV involvement was reported using echocardiography in the tricuspid annular plane showing RV involvement in 54% of Nigerian patients (Karaye 2011). They concluded that CMR can identify a broader phenotype than simply LV dysfunction. They noted that the inclusion of a control group of healthy early post-partum patients would have been ideal. Another potential weakness is the failure of their report to provide pair-wise cardiac functions (LVEF, RVEF) for each of the 34 patients. Unlike FDCM where heart failure develops over a protracted period thereby allowing LV to result in secondary RV failure, failure in PPCM patients is mostly acute.

## A protocol for collecting samples from an explanted heart

Although PPCM was first discovered in the 1800s, our understanding of the underlying aetiology of PPCM remains elusive. We propose that a combined proteomic-transcriptomic approach to examine a large as possible cohort of PPCM patients is required. However, acquiring a large cohort of PPCM tissue samples has been a challenge, due in part to the rarity of the disease. The pooling of existing PPCM samples from international heart tissue banks will yield about 30–40 PPCM patient LV samples, sufficient to establish sample variance and to estimate the number of patients required. Further international collaboration will be needed to substantially increase these numbers. Identifying new sources of PPCM samples will only be useful if the quality of the sample collection and preservation is comparable.

Here is what we do. We prepare for collecting a transplanted heart well in advance, so when we are called by the transplant coordinator,^1^ we spring into action. Briefly, while the patient is prepared to receive a healthy donor heart, we confirm there is patient consent and collect the relevant clinical data. Once the patient is connected to the perfusion machine that oxygenates the blood and returns it to the patient, the failing heart is ready to be removed (usually 40–60 min after the chest is opened). The failing heart is quickly removed so we can immediately begin isolating small (~ 1 g) samples. We start by removing a ~ 1 cm wide strip from the LV anterior free wall which is cut it into 1-cm sections from base to apex and immediately snap-frozen in liquid nitrogen. This processing of LV strips is repeated until we have collected 25–30 cryovials (1.8 ml), each labelled with a printed label to identify the sample number, heart chamber, and a unique de-identified patient code. We then collect about 10 more cryovials of tissue from the RV, at least 10 from the interventricular septum, several vials from the papillary muscles, and finally 3–5 vials from each the left and right atria. With a two-person team, it is possible for one of us to dissect the major coronary arteries (L main, LAD, circumflex and R coronary) which are easily identified on the surface of the heart. When 40 min has passed after crossclamp time, collection ceases. The remainder of the heart is placed into formalin to be collected by anatomical pathology so a report can be prepared. We have collected heart samples from about 400 heart transplant patients. We have also collected 110 donor hearts (aged from 3 months through 65 years) that were not able to be matched to patients waiting for a donor heart. The donor hearts come from patients that were declared to have suffered “brain death”, usually as a result of massive cerebral artery haemorrhage, but whose heart continued to beat. These hearts were removed from coordinator-certified donor patients. The donor hearts were perfused with sterile ice-cold cardioplegic solution that arrested contraction, packed in ice and transported to St Vincent’s Hospital, often by private jet from around Australia. The donor hearts were processed as described above except that the entire heart was used, usually resulting in > 100 vials per heart. Most of the hearts from healthy babies and children arose from swimming pool-drownings (dos Remedios et al. 2017).

In a report of the long-term outcomes for 1,938 PPCM transplant patients and 28 age-match controls, Bouabdallaoui et al. (2018) concluded that the outcomes “were favourable”, and this is supported by the experience at the St Vincent’s Hospital Heart & Lung Transplant Unit. Unfortunately, the Bouabdallaoui group did not collect tissue from these failing explanted hearts. More recently, Rasmusson et al. (2012) reported that of 42,406 transplantations, 9,419 were women and 485 of these had PPCM (Rasmusson et al. 2012). These patients had a higher list status and were younger, but graft survival was lower than in comparison to other women. In the USA, 1,258 women received LVADs either as a bridge to recovery or to transplantation, and this is another source of ventricular tissue that may have been snap-frozen and stored (dos Remedios et al. 2017). If even a small fraction of these were snap-frozen, they would be valuable for research.

We therefore need to look for existing (and future) sources of PPCM transplanted hearts. The registry of the International Society for Heart and Lung Transplantation (Taylor et al. 2007) reported that about 5,000 heart transplantation procedures have been performed annually.

Another potential source of tissue is formalin-fixed paraffin-embedded (FFPE) tissue. While this is not the ideal source for RNA sequencing because formalin fixation degrades RNA and crosslinks it to its ligands, the 10 × Genomics FFPE Visium slides employ clever chemistry to retrieve and immobilise mRNA and visually connect it to the cells of origin. The current technology has a spatial resolution of 55 µm but in 2022 Visium slides will be released that have a resolution of about 5 µm, sufficient to examine the individual nuclei of human cardiomyocytes (see 10x Genomics 2020).

## RNAseq collaboration

As a collective, we have agreed to contribute LV and RV tissue samples to undertake RNAseq analysis from PPCM patients and compared them to tissue-type matched healthy female donor hearts provided by the Sydney Heart Bank (Li et al. 2019), Ken Campbell’s tissue bank at the University of Kentucky (McDonald et al. 2020) and Zolt Arany at the University of Pennsylvania (Arany and Elkayam 2016). Collectively, we have about 30 snap-frozen samples from failing PPCM transplant patients, sufficient to establish the sample variance for the genes list in Table 2. We will continue our global search for PPCM heart samples (they are readily transported internationally in nitrogen vapour dewars that maintain − 196 °C for a week). Our aim is to achieve a tissue cohort of up to 100 hearts to achieve single cell RNAseq using the 10xVisium slide system. Tissue samples of 10–50 mg are sufficient to achieve this kind of RNAseq.

## Mass spectrometry and PPCM

If RNAseq has a failing, it is because it usually begins with a few milligrammes of tissue and yields data based on the aggregated RNA content of all the cells in the sample, including cardiomyocytes, endothelial cells fibroblasts and other resident or transient cells in the sample. In other words, it is not cardiomyocyte specific. Mass spectrometry (MS) provides information about the specific nature and modification status of the proteins (Aebersold and Mann 2016). “Bottom-up” MS and has similar limitations (Gregorich et al. 2014). Shot-gun MS requires trypsin digestion of the heart proteins, but since so many of the proteins listed in Table 2 involve truncation mutations, and several involve post-translation modification, making it nearly impossible to detect if some of these proteins might be truncated by mutations. Accordingly, our collaborator Professor Ying Ge will combine her expertise in cutting-edge high-resolution “top-down” MS-based, proteomics and functional studies to quantify intact proteins including their post-translational modifications of most sarcomeric proteins (Tucholski et al. 2020) to samples from PPCM patients.

## Track record of collaboration

When the senior author began collecting tissue samples from heart transplant patients’ hearts in 1989 with encouragement of the late Dr Victor Chang, we realised that alone we could not expect to understand the molecular nature of human heart failure without the help and expertise of the many colleagues and friends. By sharing failing and non-failing human heart tissue with a considerable number of these researchers, we have made some interesting discoveries and some real progress. So far, we have published about 160 papers (see Supplementary of dos Remedios et al. 2017) only one of which (Bollen et al. 2017) specifically addressed the role of titin mutations in the pathogenesis of PPCM.

## Is there a “cure” for PPCM and other inherited cardiomyopathies?

In most instances, the precursor of a “cure” requires an understanding of the molecular basis of the disease. In the case of hypertrophic cardiomyopathy (HCM), it was the understanding that depletion of myosin cross-bridges in the “super relaxed state” (McNamara et al. 2015) led to the realisation that the drug mavacamten stabilises the super relaxed state (Anderson et al. 2018) and restores the heart to normal activity.

In the case of PPCM, Bollen et al. (2017) were searching for a mechanism to explain why DCM patients progressively deteriorated without recovery, compared to PPCM patients that either recovered or rapidly deteriorated. They compared six cases of DCM, four cases of ischemic heart disease and four cases of PPCM with 16 age- and sex-matched healthy donor hearts and found that only in the PPCMs was length-dependent activation significantly impaired, which they attributed to reduced protein kinase A (PKA) activity.

Then more recently, Fomin et al. (2021) from a large group of German laboratories (including Wolfgang Linke) and from the Karolinska laboratory produced human induced pluripotent stem cell-derived cardiomyocytes (hiPSC-CMs). They compared wild-type controls to CMs with either a patient-derived A-band-*TTNtv* truncation mutant (*TTNtv*) or a CRISPR-Cas9-generated M-band *TTNtv*. The amount of truncated TTNtv protein increased in proportional to the inhibition of proteasomal activity. However, in the engineered hiPSC-CMs the depressed contractility due to the TTNtv could be reversed by correcting the mutation using CRISPR-Cas9 which eliminated the truncated TTN and raised the level of wild-type protein, thus restoring function. This exciting paper is more promising than anything reported so far.

## Open invitation

The authors of this review issue an open invitation to clinical research groups to collaborate with us so we can collaborate by assembling a large cohort of tissue from PPCM to better understanding of why the hearts decline and fail.

## Data Availability

Not applicable.

## Footnotes

1 Usually at 1 am.
